# Clash of kingdoms or why *Drosophila *larvae positively respond to fungal competitors

**DOI:** 10.1186/1742-9994-2-2

**Published:** 2005-01-27

**Authors:** Marko Rohlfs

**Affiliations:** 1 Zoological Institute, Department of Animal Ecology, Christian-Albrechts-University of Kiel, Am Botanischen Garten 1-9, D-2408 Kiel, Germany

## Abstract

**Background:**

Competition with filamentous fungi has been demonstrated to be an important cause of mortality for the vast group of insects that depend on ephemeral resources (e.g. fruit, dung, carrion). Recent data suggest that the well-known aggregation of *Drosophila *larvae across decaying fruit yields a competitive advantage over mould, by which the larvae achieve a higher survival probability in larger groups compared with smaller ones. Feeding and locomotor behaviour of larger larval groups is assumed to cause disruption of fungal hyphae, leading to suppression of fungal growth, which in turn improves the chances of larval survival to the adult stage. Given the relationship between larval density, mould suppression and larval survival, the present study has tested whether fungal-infected food patches elicit communal foraging behaviour on mould-infected sites by which larvae might hamper mould growth more efficiently.

**Results:**

Based on laboratory experiments in which *Drosophila *larvae were offered the choice between fungal-infected and uninfected food patches, larvae significantly aggregated on patches containing young fungal colonies. Grouping behaviour was also visible when larvae were offered only fungal-infected or only uninfected patches; however, larval aggregation was less strong under these conditions than in a heterogeneous environment (infected and uninfected patches).

**Conclusion:**

Because filamentous fungi can be deadly competitors for insect larvae on ephemeral resources, social attraction of *Drosophila *larvae to fungal-infected sites leading to suppression of mould growth may reflect an adaptive behavioural response that increases insect larval fitness and can thus be discussed as an anti-competitor behaviour. These observations support the hypothesis that adverse environmental conditions operate in favour of social behaviour. In a search for the underlying mechanisms of communal behaviour in *Drosophila*, this study highlights the necessity of investigating the role of inter-kingdom competition as a potential driving force in the evolution of spatial behaviour in insects.

## Background

A common idea in animal ecology is that adverse or stressful environmental conditions facilitate the evolution of social behaviour [1]. The formation of groups across a huge number of animal taxa is thus considered to have broad implications for the benefit of individuals, including mate finding, the efficient location and use of resources, thermoregulation, energetic benefits and defence against natural enemies or competitors [2,3]. Basic proximate prerequisites for communal behaviour are cues indicating the location of conspecifics and the ability to receive and process information regarding these cues, which in turn induce inter-individual attraction [3]. Because the costs and benefits of communal behaviour typically vary with environmental conditions, the degree to which individuals are mutually attracted is regulated by signals indicating the presences of predators, food availability, etc. [4].

**Figure 1:**
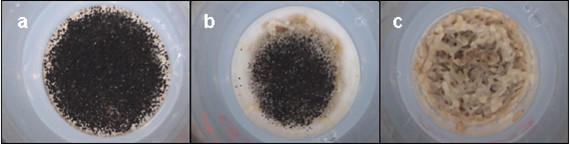
**The effect of larval density on mould growth. **The effect of *Drosophila *larval density (a. one larva, b. 5 larvae, c. 10 larvae) on the growth of *Aspergillus niger*. Patches (2.5 cm diameter) contained standard *Drosophila *rearing medium. Photographs were taken 10 days after infection with fungal spores. Spores and fly larvae were simultaneously transferred to the patches. Whereas one larvae did not significantly hamper mould development (a), five and ten larvae caused a substantial reduction in fungal growth (b) or even entirely suppressed fungal development (c). (unpublished study)

In the vast group of insects that depend on ephemeral resources, such as decaying plant tissues, dung and carrion, aggregation in the immature stages across resource patches is the result of the choice of a female to lay batches of eggs and/or to aggregate with conspecifics [5-8]. In studies of *Drosophila *as an ecological model system, one benefit that females flies seem to achieve by this spatial aggregation is that larval survival probability to the adult stage is highest at intermediate densities [9,10], indicating the existence of so-called Allee effects [11]. Competing filamentous fungi co-occurring with *Drosophila *larvae on the same patches have been demonstrated to cause high rates of mortality when larvae feed solitarily or in small groups, whereas larger groups are able to hamper mould growth [12] (Fig. 1), which in turn increases larval survival [9,13,14]. Although the mechanisms leading to mould suppression are not fully understood, physical damage of the fungal tissue from the feeding (shovelling food with the mouth hooks) and locomotor (crawling and digging) behaviour of the fly larvae [15] seems to be the major cause of the repression of mould growth [12,14].

Given the relationship between spatial oviposition patterns, Allee effects and the suppression of mould, spatial aggregation in *Drosophila *can be interpreted as an adaptive behaviour against competing fungi on larval feeding sites in order to enhance offspring survival. These ecological interrelationships might set conditions for facilitating social behaviour in the fly larvae because, at the level of larval behaviour, a more efficient strategy that might control the rapid establishment of noxious fungi would be to exert physical stress directly on fungal colonies. Thus, larvae should display an assortative behaviour on the site on which fungi are growing, rather than moving randomly and independently of each other across a resource patch, by which the fungal tissues might only incidentally be destroyed. In the present study, I have provided groups of *Drosophila melanogaster *Meigen (Diptera, Drosophilidae) larvae with fungal-infected (2-day-old colonies of *Aspergillus niger *van Tieghem) and uninfected (control patches) (F-C treatment) food patches and examined whether the distribution of larvae across the patches is driven by fungal infection. In comparison with this naturally occurring heterogeneity in patch quality, I have also studied the distribution of fly larvae when they were offered only infected (F-F treatment) or uninfected (C-C treatment) food patches in order to test for the existence of grouping behaviour in two types of homogenous larval environment. If grouping is irrelevant under the given experimental setting, no deviation from the regular larval distribution across the food patches would be expected, i.e larvae should distribute themselves across patches in order to minimise larval competition for food [16].

Although *Drosophila *is a thoroughly studied model organism in foraging biology [17,18], knowledge about social interactions between the insect larvae is surprisingly limited. This is intriguing because drosophilids are also model organisms in spatial ecology in which *Drosophila *communities are characterised by strong intraspecific aggregation across patchily distributed substrates (e.g. decaying plant tissues) [19-21]. The lack of knowledge concerning social interactions among larvae and its possible role in competition with filamentous fungi have provided the specific impetus of the present study.

## Results

### Larval aggregation in the F-C treatment (Δ*pl*)

**Figure 2:**
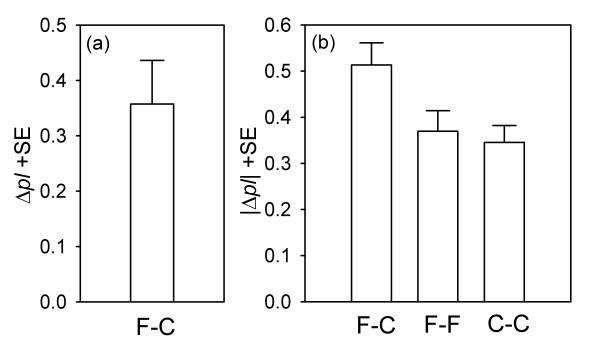
**Larval aggregation in the heterogeneous (F-C) and two types of homogeneous (F-F and C-C) larval environment. **(a) Δ*pl *(where Δ*pl *= proportion of larvae from the fungal-infected patch – proportion of larvae from the uninfected patch) as a measure of larval aggregation in the F-C treatment (Δ*pl *= 0: no effect of fungal-infected patches on larval distribution behaviour; Δ*pl *> 0: aggregation of larvae on fungal infected patches; Δ*pl *< 0: larvae avoid fungal colonies). (b) |Δ*pl*| as a measure of the general tendency of *Drosophila *larvae to aggregate with conspecifics in the heterogeneous environment (F-C) and two types of homogeneous environment (F-F and C-C). Because larval aggregation in the F-C treatment was measured independently of the patch type (see Methods), |Δ*pl*| is larger than Δ*pl *(2a). (F: fungal-infected patches, C: uninfected control patches)

The proportion of larvae on fungal-infected patches minus the proportion on uninfected patches, Δ*pl*, was used as a measure of the way in which *Drosophila *larvae distributed themselves between the two types of food patches in the F-C treatment (see method section for details). The number of larvae in both food patches (LARVAE) and the experimental day (DAY) did not influence Δ*pl *(Table 1). The estimated intercept for Δ*pl *was significantly different from zero (GLM *d.f. *= 1, *mean square *= 4.475, *F *= 20.13, *P *< 0.0001, *N *= 35), the positive value for Δ*pl *(Fig. 2a; *intercept estimate*: 0.3576 ± 0.0797, *t *= 4.49, *P *< 0.001) indicating the aggregation of larvae on fungal-infected sites (see method section).

**Table 1:** Effect of LARVAE and DAY on larval aggregation in the F-C treatment. Analysis of variance for the effect of the number of larvae in both food patches (LARVAE) and experimental day (DAY) on *Drosophila *larval distribution between fungal-infected and uninfected food patches (F-C treatment).

**Explanatory variable**	***d.f.***	**Mean square**	***F*-value**	***P***
LARVAE	1	0.0361	0.15	0.7042
DAY	3	0.0470	0.19	0.9018
Error	30	0.2462		

### Comparison of larval aggregation in the F-C, F-F and C-C treatment (|Δ*pl*|)

|Δ*pl*|, the absolute value of Δ*pl*, was used as a measure of the general tendency of *Drosophila *larvae to aggregate with conspecifics in the heterogeneous environment (F-C) and the two types of homogenous environment (F-F or C-C). By using |Δ*pl*|, aggregation in the F-C treatment was quantified independently of whether a food patch was infected with fungi or not. With regard to all three larval environments, the estimated intercepts for |Δ*pl*| were significantly different from zero, and hence indicate larval aggregation (Table 2). Within each treatment LARVAE and DAY had no effect on |Δ*pl*| (Table 3). In comparison with the homogenous environments (F-F and C-C treatment), the F-C treatment induced stronger larval aggregation (Fig. 2b, Table 4). Moreover, there is a statistical trend of LARVAE influencing fly larval aggregation (Table 4). This was due to differences in LARVAE as a function of TREATMENT (GLM *d.f. *= 2, *mean square *= 0.0134, *F *= 3.34, *P *= 0.0393, *N *= 105). Significantly fewer larvae were found to be feeding in both patches in the C-C treatment (8.89 ± 1.64 SE) than in the F-C (9.43 ± 0.95 SE) or the F-F treatment (9.46 ± 0.61 SE). However, LARVAE within one type of environment had no effect on larval aggregation (Table 3).

**Table 2:** The general tendency to aggregate with conspecifics (|Δ*pl*|) in the heterogeneous (F-C) and two types of homogeneous (F-F and C-C) larval environment. Test of the effect of intercept as the only explanatory variable for the general tendency of *Drosophila *larvae to aggregate with conspecifics (measured as |Δ*pl*|, see text for details) in three types of larval environment (F-C, F-F or C-C). Whereas |Δ*pl*| = 0 and no explanatory power of intercept would indicate a regular distribution of larvae across the food patches, |Δ*pl*| > 0 and a significant effect of intercept indicates larval aggregation in one of the experimental food patches (see also Fig. 2b). Note that, in contrast to Δ*pl *(Fig. 2a), |Δ*pl*| measures larval aggregation in the F-C treatment independently of whether a food patches was fungal-infected or not. For each type of larval environment an individual test was performed, with *N *= 35 for each treatment.

	***Parameter estimate***		
**Larval environment**	**Intercept ± SE**	***t*-value**	***P***

F-C	0.51 ± 0.05	10.55	<0.0001
F-F	0.37 ± 0.05	8.13	<0.0001
C-C	0.35 ± 0.04	9.24	<0.0001

**Table 3:** The effect of LARVAE and DAY on the general tendency of *Drosophila *larval aggregation (|Δ*pl*|) under three environmental conditions. Analysis of variance for the effect of LARVAE and DAY on *Drosophila *larval distribution between food patches in three different larval environments (F-C, F-F or C-C).

**Larval environment**	**Explanatory variable**	***d.f.***	**Mean square**	***F*-value**	***P***
F-C	LARVAE	1	0.2120	2.63	0.1154
	DAY	3	0.0931	1.15	0.3433
	Error	30	0.0807		

F-F	LARVAE	1	0.0682	1.03	0.3177
	DAY	3	0.1132	1.71	0.1855
	Error	30	0.0661		

C-C	LARVAE	1	0.0329	0.66	0.4236
	DAY	3	0.2090	4.18	0.4998
	Error	30	0.0400		

**Table 4:** The effect of the larval environment on the general tendency of *Drosophila *larvae to aggregate with conspecifics (|Δ*pl*|). Mixed model analysis of variance for the tendency to aggregate with conspecific larvae in *D. melanogaster *in three types of larval environment (F-C, F-F or C-C). Larval aggregation was measured as |Δ*pl*|, the absolute value of Δ*pl *(see Methods). TREATMENT (F-C, F-F or C-C) and LARVAE were fixed main effects, whereas experimental day (DAY) was a random factor. DAY is nested within TREATMENT and was used as the error term in testing the effect of TREATMENT.

**Explanatory variable**	***d.f.***	**Mean square**	***F*-value**	***P***
TREATMENT	2	0.4168	5.08	0.0302
LARVAE	1	0.2237	3.44	0.0670
TREATMENT (DAY)	9	0.0831	1.28	0.2603
Error	92	0.0651		

## Discussion

On the background of ecological interactions between insects and filamentous fungi on ephemeral resources, the experiment presented in this study was designed to test for social attraction in *Drosophila *larvae, an attraction that I hypothesised to be advantageous when larvae are confronted with noxious moulds. The results demonstrate that the fly larvae significantly aggregated on food patches on which young fungal colonies were growing (F-C treatment, Fig. 2a). Moreover, when provided with a homogeneous environment (F-F or C-C treatment), larvae displayed significant aggregation across the two food patches (Fig. 2b). In comparison, however, aggregation was significantly enhanced when larvae had the choice between a mould-free and a mould-infected site (Fig. 2b). Thus, the results suggest that grouping behaviour in *Drosophila *larvae involves both mutual attraction between group members [2] and the attraction of individuals to the same environmental stimulus [22], i.e. cues emitted by the fungi.

Group formation in eusocial insects and those living in groups for part or most of their lives is often mediated by pheromones, e.g. cuticular hydrocarbons that induce attraction between individuals [22,23]. Chemical communication is also widespread in drosophilid behaviour, including those associated with spatial aggregation in adult flies [24-26]. Because several receptors on the cephalic lobe of *Drosophila *larvae have gustatory, mechanosensory and olfactory functions [27], both chemical and physical cues (e.g. substrate vibrations caused by larval movements) might be involved in mutual attraction. However, the mechanisms leading to grouping and group cohesion in *Drosophila *larvae are unknown. Interestingly, strong social attraction (communal digging) between *Drosophila *larvae is present in third-instar larvae, a behaviour regulated by a peptide neuromodulator (*Drosophila *neuropeptide F, dNPF) [28]; this shows striking similarities to the correlation of social feeding and the expression of a neuropeptide Y receptor homologue (NRP-1) in *Caenorhabditis elegans *[29]. Moreover, neurons that detect aversive environmental stimuli have been demonstrated to induce social feeding in *C. elegans *[30], thus providing support for the proposed relationship between environmental stress and social behaviour [31]. In contrast to *C. elegans*, the intimate communal digging behaviour in old third-instar *Drosophila *larvae does not occur in the context of food foraging behaviour but is part of the post-feeding phase prior to pupation [28]. Whereas downregulation of dNPF expression coincides with social behaviour in old and non-feeding *Drosophila *larvae, higher levels of dNPF expression in younger larvae seem to suppress strong larval aggregation and communal digging behaviour [28]. Therefore, it remains to be seen whether similar neural regulatory mechanisms are involved in earlier developmental stages of *Drosophila *larvae with respect to social affinity related to foraging for food and attraction to fungal competitors. With regard to the proximate causes of attraction to mould-infected sites, fungal-borne volatiles such as CO_2 _or ethylene [32] might be perceived by fly larvae and might guide them to young mould colonies.

A general reason for the formation of social groups seems to be an adaptive response to stressful environmental conditions [1,31]. As outlined in the introduction, filamentous fungi co-occurring with drosophilids on larval feeding sites can impede fly larval development; indeed, larval aggregations have been shown successfully to suppress mould growth [12] (Fig. 1). Consequently, the presence of fungi may indicate stressful ecological conditions that initiate attraction towards fungal patches and enhance the mutual attraction of *Drosophila *larvae (Fig. 2). In connection with the benefits that accrue from mould suppression, the present study demonstrates that the larval-driven inhibition of fungal development is not a mere by-product attributable to the maternal decision to aggregate eggs across patches but is the consequence of a positive response of individuals to conspecifics. Because adult density-dependent oviposition choices influence a larva's food quality and its susceptibility to natural enemies [33] or abiotic stress, as well as its probability of coming into contact with intra- and interspecific competitors, the present study demonstrates the possibility of adaptive behavioural relationships between the well-known adult gregariousness in *Drosophila *and communal behaviour in the immature stages. Further investigating this kind of behavioural adult-offspring correlations would strongly contribute to our understanding of the evolutionary costs and benefits of spatial aggregation in insect communities [34].

## Conclusion

The study presented here demonstrates that fungal-infected food patches (1) attract first-instar *Drosophila *larvae and (2) enhance group foraging behaviour. Because the larval-driven reduction in mould growth [12] has been shown to improve the chance of larval survival to the adult stage [9,13,14], social attraction to fungal-infected sites may reflect an adaptive behavioural response. In connection with the maternal behaviour of aggregating eggs across substrate patches, the condition-dependent mutual attraction of larvae can be discussed as a communal defence behaviour against competing mould. Given that filamentous fungi seriously deteriorate the developmental conditions for insect larvae, mould may constitute one important ecological factor that has, at least in the larval stages, facilitated social attraction in *Drosophila*. Thus, this study highlights the largely unappreciated role of inter-kingdom competition as a potentially important driving force in the evolution of insect behavioural traits. In general, the group formation of *Drosophila *larvae in response to well-defined ecological conditions might be an interesting model system for the study of proximate and ultimate aspects of social biology.

## Methods

### Experimental set-up

I experimentally analysed the grouping behaviour of *Drosophila *larvae using a *D. melanogaster *strain that originated from wild animals caught in 2003 near Kiel, northern Germany (approx. 54° N, 10° E). Flies had been reared for 18 generations on standard *Drosophila *medium (30 gram corn meal, 30 gram sugar, 30 gram brewer's yeast extract (Leiber, Germany), and Nipagin) under constant environmental conditions (photoperiod of 16 hours and a temperature of 22°C). In order to obtain first instar larvae, a population of approx. 300 individuals (five to seven days old) were offered a 10 cm Petri dish containing a hard Agar medium (22 gram Agar, 90 cm^3 ^sugar beet syrup and 9.5 cm^3 ^Nipagin (10% in 95% ethanol) per 500 cm^3 ^water), on which they were allowed to oviposit for a period of 16 to 18 hours, including a period of darkness. Subsequently, flies were removed and the eggs were incubated at 22°C over a 16-hour photoperiod. After 24 hours, almost all larvae had hatched from the eggs and were then isolated from the medium by washing them off the Agar plate onto fine-meshed gauze with water. These larvae were used in the experiments.

I used the same medium as for fly rearing (without adding the antifungal agent Nipagin) to simulate an uninfected or a fungal-infected larval environment as follows: aliquots of 3.5 cm^3 ^hot medium were transferred to each of two small pots (10 mm diameter, 5 mm high) that were glued to the bottom of a Petri dish (45 mm diameter, 13 mm high). Within one Petri dish, the larval food patches were placed at a distance of approx. 10 mm and surrounded by an Agar layer (5 mm high), so that the surfaces of the Agar and the food patches were at the same level. After the food patches had cooled down, one patch was provided with 1 μl of water containing approx. 800 conidiospores of the fungus *A. niger *(F treatment) and, as a control, the second patch was provided with spore-free water (C treatment). In addition to this F-C treatment, I simultaneously prepared arenas in which both patches were infected with spores (F-F) or both patches remained uninfected (C-C). The arenas were immediately sealed with lids and incubated under the aforementioned conditions. Two days later, the fungal spores had germinated and tiny translucent hyphal colonies were visible. A group of ten *Drosophila *larvae were transferred, with a fine brush, to each arena at a distance of approx. 10 mm from each of the two food patches. Subsequently, the arenas were sealed with lids and stored at 22°C in an illuminated incubator. In order to avoid any systematic effects on larval distribution behaviour that might be caused by the position of the light tubes, the arenas of all three treatments were randomly arranged in the incubator. After six hours, the number of larvae in each food patch was recorded. Preliminary experiments had shown that this time period was sufficient to obtain final larval distribution patterns across the two patches, which remained nearly constant until the next day. Five to ten replicates for each treatment were simultaneously prepared at four different days.

### Statistical analysis

Based on the number of larvae that were found to be feeding in both patches, I calculated the proportion of larvae in each patch. Larvae that were not found on any of the food patches were ignored. However, I tested for the effect of the number of larvae in both patches (LARVAE) on the degree to which larvae aggregated across the food patches (see below). To obtain a measure of the degree of larval aggregation across the two food patches in the F-C treatment, the proportion of larvae on the fungal-infected patch in each arena was reduced by the proportion of larvae on the uninfected patch, yielding Δ*pl*. Δ*pl *= 0 would indicate no effect of the presence of fungal-infected and uninfected food sites on larval distribution patterns. Whereas Δ*pl *> 0 would indicate aggregation on fungal-infected sites and thus larval attraction to fungal colonies, Δ*pl *< 0 is expected if larvae avoid fungal colonies and aggregate on uninfected patches. Subsequently, I used the absolute values of Δ*pl*, |Δ*pl*|, that were obtained in all three types of treatment (F-C, F-F and C-C) in order to compare the tendency to aggregate with conspecific larvae in the heterogeneous larval environment (F-C) with larval aggregation in two types of homogeneous environment (F-F or C-C). Note that, because the absolute value of Δ*pl *can only be equal to or larger than zero, |Δ*pl*| measures larval aggregation in the F-C treatment independently of whether a food patch was fungal-infected or not.

I applied the GLM procedure provided by SAS version 8.2 to test if *Drosophila *larvae aggregated on fungal infected food patches, i.e. if Δ*pl *is significantly larger than 0 (see above). For this only the intercept was tested as an effect in the statistical model [35]. The result of the parameter estimate for the intercept are given. Before this test, I verified that LARVAE and experimental DAY did not affect Δ*pl *(Table 1), which justifies the removal of these variables from the full model (backward elimination of non-significant variables) [36]. The same procedure was applied to test for the general tendency of larval aggregation (measured as |Δ*pl|*) under different environmental conditions (see Table 2 to 4).

To analyse the effect of TREATMENT (F-C, F-F or C-C) and LARVAE on the general propensity of *Drosophila *larvae to aggregate across the experimental food patches (|Δ*pl*|), I used the aforementioned GLM procedure with a RANDOM statement to account for possible effects of experimental DAY on larval distribution patterns. In this model, TREATMENT and LARVAE were fixed main effects. Since five to ten replicates for each treatment were prepared at four different days, DAY was considered as a categorical random factor. DAY is nested within TREATMENT and was used as the error term in testing for the effect of TREATMENT [37]. The results of the tests of hypotheses for mixed model analysis of variance are shown in Table 4.
